# Reanalysis and Simulation Suggest a Phylogenetic Microarray Does Not Accurately Profile Microbial Communities

**DOI:** 10.1371/journal.pone.0033875

**Published:** 2012-03-22

**Authors:** David J. Midgley, Paul Greenfield, Janet M. Shaw, Yalchin Oytam, Dongmei Li, Caroline A. Kerr, Philip Hendry

**Affiliations:** 1 Division of Food and Nutritional Sciences, CSIRO, North Ryde, New South Wales, Australia; 2 Division of Mathematics, Informatics and Statistics, CSIRO, North Ryde, New South Wales, Australia; Argonne National Laboratory, United States of America

## Abstract

The second generation (G2) PhyloChip is designed to detect over 8700 bacteria and archaeal and has been used over 50 publications and conference presentations. Many of those publications reveal that the PhyloChip measures of species richness greatly exceed statistical estimates of richness based on other methods. An examination of probes downloaded from Greengenes suggested that the system may have the potential to distort the observed community structure. This may be due to the sharing of probes by taxa; more than 21% of the taxa in that downloaded data have no unique probes. In-silico simulations using these data showed that a population of 64 taxa representing a typical anaerobic subterranean community returned 96 different taxa, including 15 families incorrectly called present and 19 families incorrectly called absent. A study of nasal and oropharyngeal microbial communities by Lemon et al (2010) found some 1325 taxa using the G2 PhyloChip, however, about 950 of these taxa have, in the downloaded data, no unique probes and cannot be definitively called present. Finally, data from Brodie et al (2007), when re-examined, indicate that the abundance of the majority of detected taxa, are highly correlated with one another, suggesting that many probe sets do not act independently. Based on our analyses of downloaded data, we conclude that outputs from the G2 PhyloChip should be treated with some caution, and that the presence of taxa represented solely by non-unique probes be independently verified.

## Introduction

Understanding the structure and function of microbial communities is critical as they play key roles in environmental processes such as nutrient cycling [1]. Molecular biology has delivered numerous techniques that have revolutionised the field of microbial ecology. The most recent, high-throughput sequencing technologies have resulted in quantum leaps in our understanding of these communities [2]. For highly replicated experiments or for environmental monitoring, however, massive sequencing can still be prohibitively expensive. Microarray technologies like the PhyloChip [3] or Geochip [4] – which are designed to detect bacteria and archaea in the environment using 16S ribosomal DNA or functional genes, respectively – provide an affordable alternative.

The PhyloChip is widely regarded as an innovative technology that offers great potential for environmental research and has won numerous accolades [5]–[7]. The technology has been used to assay microbial diversity in habitats including soil, sediments, plant tissues and air along with various human microbiomes [3], [8]–[15]. The second generation (G2) of this technology is designed to detect over 8700 microbial taxa in environmental samples. The array is based on 25 base pair single-stranded DNA probes, derived from the 16S ribosomal DNA, which are bound to a silicon chip. Labelled target DNA is washed across the chip, matching DNAs bind to the probes, and are detected by fluorescence. Each perfectly matched (PM) probe is accompanied by a mismatched (MM) probe in which the central nucleotide is replaced with one of the 3 alternate nucleotides. Taxa are represented on that array by a set of at least 11 probes. The detection of the OTUs (≈species) on the G2 microarray, occurs when a specified percentage of the probes (typically 90 to 95%) within a probe set are positive, ie intensity of the PM probe is at least 1.3 times that of the MM probe.

## Results

After obtaining PhyloChip G2 OTU numbers from PhyloTrac [16], we obtained the corresponding perfectly matched probe set data from the Greengenes web site (http://greengenes.lbl.gov/cgi-bin/nph-show_probes_2_otu_alignments.cgi) as directed in Brodie et al [17]. In that data, (Data S1) we identified a total of 521,206 PM probes in 8934 probe sets. Our subsequent analyses are based on this data set, and the simulated microarray based on this data we will call the *In Silico Phylogenetic MicroArray* (ISPMA). Since the target 16S rDNA is highly conserved, many of the probes are shared between probe sets [3]. There were 182,653 different DNA sequences, of which 159,824 occurred only once and were hence unique. The remaining 22,829 probes occurred in at least 2 and up to 300 probe sets. On average, probe sets contain 58 probes, though probe sets as large as 762 probes were detected. We found 222 probe sets with 10 or fewer probes. These 222 small probe sets are usually not included in analyses [3] reducing the effective number of OTUs to 8712, slightly fewer than the 8741 reported in Brodie et al [3], the difference probably being due to our use of PhyloTrac to obtain OTU numbers. The PhyloChip has been reported to contain 297,851 probes, of which half are mismatch probes, thus there are approximately 148,925 matched probes on the microarray [18]. The difference between this number and the 182,625 different probe sequences we identified in the Greengenes database may be large probe sets designed for pathogen-specific detection [19] not typically used for environmental samples.

**Figure 1:**
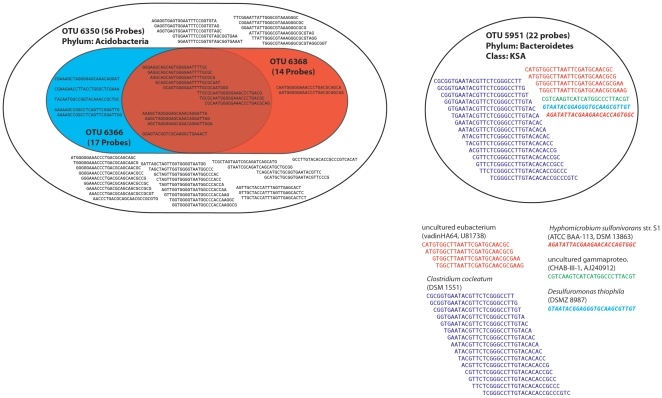
Two examples of probe sharing between OTUs. On the left are shown the 56 probes that represent the OTU 6350. Probes shared with OTU 6368 are shown in the red shape, while those shared with OTU 6366 are shown in blue. The intersection of the red and blue shapes shows probes shared by all three OTUs (centre circle). All 14 and 17 probes (100% of the probe sets) for 6368 and 6366, respectively, can be found in the probe set for OTU 6350. On the right is illustrated how the complete 22 probe, probe set for OTU 5451 can be assembled from probes representing other OTUs.

Of the 8712 probe sets in the downloaded data with greater than 11 probes, 21.4% (1864) contain no unique probes (Table S1). That is, the entire probe set can be found within the probe sets of other taxa. In broad terms, these 1864 OTUs can be divided into two groups. The first comprises those OTUs whose probe sets are exact subsets of other single organisms (Fig. 1). This means that if a particular organism is actually present in a sample examined on the array, all the organisms whose probe sets are subsets of its probes will necessarily appear to be also present. For example, the probe set for the acidobacterial OTU 6350 also includes the entire probe sets for both acidobacterial OTUs 6366 and 6368 (Fig. 1). Thus, if 16S rDNA from a pure culture of 6350 was hybridised to the ISPMA, three probe sets would be detected, all perfectly matched, falsely leading the experimenter into believing that OTUs 6366 and 6368 were also present in the sample. This ‘subsetting’ phenomenon occurs in 327 OTUs, whose probe sets are exact subsets of other OTUs. In most cases, these 327 OTUs individually are subsets of one or two other OTUs, however, more extreme examples were found. For example, the probe set for OTU 1405, an *Arthrobacter* species, is a subset of some 39 other OTUs, similarly the probe set for the actinobacterial OTU 1687, *Jonesia quinghaiensis*, is an exact subset of 61 other OTUs.

The second group are those OTUs for which two or more other OTUs can be combined to complete their probe set. Thus, an OTU which is not actually present in a sample will necessarily be identified as present, if the sample contains sufficiently many of its donor OTUs (Fig. 1B). An example of this phenomenon is given in Figure 1B where all the probes in the probe set for OTU 5951, an OTU from the phylum Bacteroidetes (class KSA) can also be found in a union of other OTUs. Intriguingly, 15 of the probes are also used to detect the presence of a Firmicute (*Clostridium cocleatum*) and only one probe originated in a member of the Bacteroidetes (in our example, *Hyphomicrobium sulfonivorans*). It is worth noting that even if this Bacteroidetes taxon was absent, and the other 4 taxa present, 95.4% (21/22 probes) of the probe set would still be found, and using the standard cut-offs of 90–95%, OTU 5451 would still be deemed to be present under normal analyses.

In addition to probe sets that register presence when their targets are actually absent, we have identified over 500 erroneous probe sets in the downloaded data that will not report presence when their targets are actually present (Table S1). There appears to be two main causes of these errors, in some cases undefined bases (Ns) in reference sequences have been deleted and the non-contiguous bases rejoined, in other cases it appears that probes may have been designed to consensus sequences.

In order to better understand how the downloaded probe sets might have been designed we plotted probe set uniqueness against probe set size (Figure S1A) and performed simulations, described in Methods S1, to try to delineate the different probabilistic characteristics present in these data (Figure S1B). Probe data downloaded from Greengenes and the simulation reveal a general trend where probe set uniqueness declines as probe set size increases.

**Figure 2:**
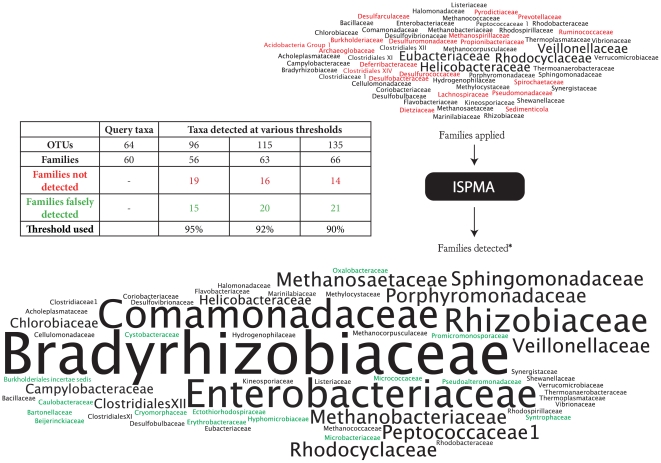
Results of the ISPMA analyses for the 64 OTU ‘sample’. The number of OTUs representing each family is proportional to the font size in this “Wordle” image. The families shown ‘pre-analyses’ (top) are represented by one (smaller text), or two (larger text) OTUs. After analyses, using a 95% detection threshold, however, some families are ‘increased’ in their OTU numbers by an order of magnitude. Families shown in red in the input sample are not present in the output set, families in green font in the output set are not present in the input sample. The inset table also shows the outputs at 90 and 92% detection thresholds.

To delve further into how the PhyloChip may be functioning, we performed an *in silico* hybridisation assuming perfect matching. To the ISPMA we presented sequences from 64 OTUs (Table S2) that were an approximation of an anaerobic microbial community and determined how many OTUs would be detected as being present using the 90, 92 and 95% thresholds (Fig. 2 and Table S3).

Our *in silico* trials indicate that in addition to inflating the number of taxa detected, the ISPMA also appears to distort the observed community structure (Fig. 2). Indeed, using the most stringent (95%) cutoff with a ‘sample’ of 64 species, the ISPMA detected a total of 96 taxa. At the family level, 19 families actually present were not detected and representatives of 15 families were incorrectly called as present. Specifically, OTUs representing the families Burkholderiaceae, Desulfurococcaceae, Desulfuromondaceae, Lachnospiraceae, Methanospirillaceae, Prevotellaceae, Psuedomonadaceae, Pyrodictiaceae and Spirochaetaceae, and others, were not detected. In contrast, OTUs from families: Bartonellaceae, Beijerinklaceae, Burkholderiales *Incertae Sedis*, Cryomorphaceae, Cystobacteraceae, Erythryobacteraceae, Micrococcaceae, Pseudoalteromonadaceae and others, were detected, despite being absent from the 64 species ‘sample’ (Fig. 2). Moreover, though only single OTUs from the Bradyrhizobiaceae and the Comamonadaceae were in the 64 OTU ‘sample’, nine and ten OTUs from these families were detected by the ISPMA (Fig. 2). When comparing the different thresholds, it is evident that while more taxa were correctly called present using the lower thresholds, the number of taxa falsely detected was even greater.

In a recent paper by Lemon et al [20], the microbial diversity present in the nose and oropharynx from seven healthy individuals was compared using both the 16S rDNA clone and sequence method and the G2 PhyloChip. The clone method identified 36 and 71 taxa in the nose and oropharynx, respectively, and statistically projected (from ∼700 clones from each site) estimates of richness (Chao 1) for each site were 50±7.2 and 120±17. In comparison, the PhyloChip detected 911 nasal and 1066 oropharyngeal taxa. There was significant overlap of taxa between the sites and a total of 1325 different taxa were detected. The majority of these were detected at low levels and their presence was not independently validated. Clearly, all methods have their shortcomings and the cloning of PCR products is likely to under-represent the real diversity through limited sampling of the clone pool as well as PCR bias. Nevertheless, of the 1325 taxa detected, about 950 are from OTUs that, in the downloaded data, have no unique probes and more than 1100 have fewer than 10% unique probes, and therefore could have been incorrectly counted as present due to the contribution of DNA from other taxa. Whilst it is not reasonable to assume that all 1100 taxa are absent from the sites in question, the remaining number of taxa, ∼225, is much closer to the number predicted by Chao 1 estimates of richness.

**Figure 3:**
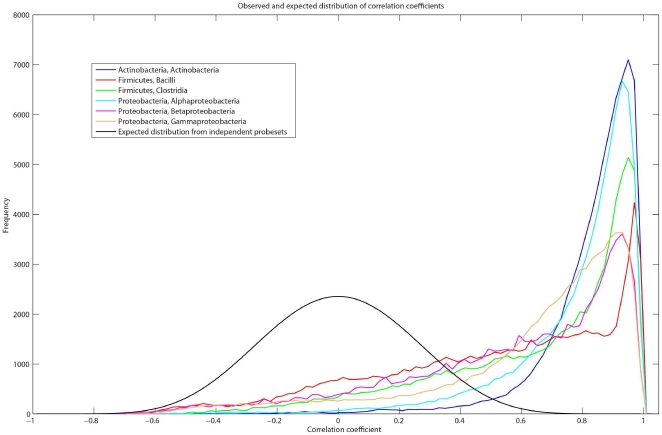
Reanalysis of some of the Texas Aerosol data by Brodie et al [**3**]. Observed and expected distribution of correlation coefficients for pairwise comparisons of intensity of detected OTUs within the classes Actinobacteria, Bacilli, Clostridia, Alpha-, Beta- and Gamma- Proteobacteria and the expected distribution of independent probe sets (black).

Since the sharing of probes is more likely to occur within phylogenetic groups, the problem of false positives would be most likely to occur within groups. In order to investigate this, we re-examined a random subset of the results of Brodie et al [3] for the classes Actinobacteria, Bacilli, Clostridia, Alpha-, Beta- and Gamma- Proteobacteria. For each pair of OTUs within a class, the OTU abundance (intensity) data from 18 different PhyloChip experiments was plotted and a Pearsons correlation coefficient (R-value) was computed. Since there are >10^5^ such plots, a histogram of these values for each class was plotted and compared to the expected distribution for independent probe sets. All six classes show a distribution of R-values that is strongly skewed towards 1 (Fig. 3). That is, the abundance data of a disproportionately large number of probe sets appear to be strongly correlated with each other, and hence are not independent. This phenomenon also occurs in other smaller-sized classes commonly detected by Brodie et al [3] including the Acidobacteria, chloroplastic sequences (Cyanobacteria), Solibacteres and the Verrucomicrobiae (data not shown).

## Discussion

Data presented here suggests that analyses using the G2 PhyloChip may be problematic. Our analysis of the data downloaded from Greengenes indicates that there were 1864 OTUs with no unique probes, 6829 OTUs with at least one shared probe and 19 OTUs with no shared probes. The presence of OTUs defined by these probe sets without unique probes and should be viewed critically if detected. It is possible for these 1864 taxa to be detected to 100% of their probe set, without the target organism being present. Moreover, papers using PhyloChip do not require 100% of probes to match in order to call a taxon as present. Instead, cutoffs of 90 to 95% are typically used which would increase the numbers of OTUs that cannot be reliably identified as present or absent in a given sample.

Using a 90, 92 or 95% cutoff, the ISPMA simulation based on downloaded data indicated an inflation of OTU richness. It is important to note that the community tested on the ISPMA were selected without any prior knowledge of how they would affect results; instead, sequences were selected to approximate a sub-surface, anaerobic microbial community. It would thus be possible to engineer greater inflation rates of OTU richness if a mixture of OTUs that contributed large numbers of shared probes were chosen, and the converse is also true. In part this observation may account for some of the significant differences observed in estimations of species richness in environmental samples between the PhyloChip and cloning and sequencing [21], [22] approaches. For example, estimates of OTU richness derived from the PhyloChip were 2 to 5 times more OTU rich than those derived from Good's-adjusted [23] clone-sequence information from uranium mine soils for the same samples [21]. Likewise, Chao 1 estimates of species richness based on clone sampling in the study of Lemon et al [20] were almost 10-fold lower than that detected by the PhyloChip.

Moreover, for the study of Lemon et al [20] zero, or low, probe set uniqueness, within the downloaded data, was predictive of the microbial diversity observed. That is, of the 1325 detected OTUs, ∼950 were present in the list of OTUs which, for the downloaded data, contained no unique probes (Table S1). The hypergeometric probability of this occurring by chance is extremely remote (P≪0.0001). Initially we hypothesized that the ISPMA simply inflated OTU richness, detecting all query taxa and then perhaps adding a small number of closely related taxa. However, it seems to distort the observed community composition further, by variously omitting taxa which are actually present, as well as adding large numbers of taxa increasing the diversity of some families (the Bradyrhizobiaceae and Comamonadaceae, in our example) by an order of magnitude. Moreover, this distortion does not necessarily require related taxa to be present in the sequences applied to the array. In our 64 taxa (95% threshold) test, members of 15 different families were falsely detected without representatives of these families being present in the query taxa.

Reanalysis of data from Brodie et al [3] indicated that a disproportionately large number of probe sets appear to be strongly correlated with each other, and were not independent. In normal microbial communities, a range of interactions between OTUs are possible including: symbiosis, commensalism, competition and parasitism. These interactions should be ‘observed’ in Phylochip data as positive or negative relationships between the abundance of pairs of OTUs. Most interactions would be expected to be neutral, resulting in no relationship (independence) between the pairs of OTUs. This was not observed and the overwhelming predominance of positively correlated abundances of OTUs within each class is likely to be due to the same probesets contributing to multiple OTUs.

On the functioning of the ISPMA, it is noteworthy that the present study has not examined the issue of cross hybridisation. This analysis is based on the best case, perfect matching scenario, and the performance of the chip may be reduced by sub-optimal hybridisation.

Regardless of potential problems highlighted here, the PhyloChip has potential to rapidly assay microbial communities at relatively low cost and we understand that these issues may have been addressed in subsequent generations of the technology. To date, we believe erroneous results generated on the G2 PhyloChip may affect approximately 50 published manuscripts or conference proceedings in the microbial ecology field. In any of these studies, we recommend investigators check for the presence of the 1864 OTUs that contain no unique probes. If detected, their presence should be critically examined. Following this, an assessment of intensity of probe set pairs, across multiple arrays, should be undertaken, checking thoroughly for co-linearity with the consideration of biological interactions within the environment. We believe that meaningful interpretation, albeit with a potentially smaller number of organisms may still be possible by careful re-analyses of those results.

## Methods

### Probe and probe set uniqueness

The OTU probe numbers were obtained from Phylotrac [16] and the corresponding aligned 16S rDNA sequences and their associated probes were downloaded from Greengenes in November, 2010 (http://greengenes.lbl.gov/cgi-bin/nph-show_probes_2_otu_alignments.cgi) as directed by Brodie et al (2006) [17]. The downloaded file was then parsed to obtain just the set of probes corresponding to each OTU. The uniqueness of these probes was determined by adding the probes to a hash table, incrementing a counter associated with each probe sequence whenever a probe was encountered multiple times. The count values from this hash table were then used to construct a uniqueness histogram, showing how many probes were used only once, how many were used in two OTUs and so on. The test for probe set uniqueness took the same file of probe sets and again stored the probes in an all-probes hash table, together with their repetition count. The code then went through each probe set, looking up each probe in the all-probes hash table and incrementing the ‘unique probes’ counter for the probe set whenever a probe had a repetition count of 1.

### Subsetting probe sets

Subsetting probe sets (probe sets where every probe can be found in a single other ‘containing’ probe set) were found by first matching each of the probes from each probe set in turn against all of the probes from all the other probe sets. A probe set was determined to be fully contained in those cases where every probe from that set matched a probe in the probe set for a single other OTU.

### In Silico Phylogenetic MicroArray and contributing probe sets

The *ISPMA* uses a set of hash tables, each one containing the probes from a single OTU. A simulated environmental sample was constructed by creating a file containing a number of reference 16S rDNA sequences (in FASTA format). This sample was ‘hybridised to’ the probe sets by turning each reference sequence into a complete set of 25-mers and looking up each of these 25-mers against each of the OTU probe set hash tables in turn. The counts of unique matches to each OTU set were accumulated over all the reference sequences and reported at the end. Probe sets where more than 90%, 92% or 95%) of the probes had matches from any of the reference sequences were then regarded as ‘present’. Examples of how unrelated organisms can share probes and contribute to the counts used to determine OTU presence also came from ISPMA process. The code that implements this process will accept a single OTU id as a ‘target’ and all matches to this OTU's probe set are written to an output file for further analysis. In order to compare phylogenetic identity of taxa before and after ISPMA analyses, input ‘samples’ and results from the ISPMA were compared using RDP classifier [24] to ensure consistency of taxonomy. Word clouds of families, used in Figure 2, were constructed using Wordle (Jonathan Feinberg, http://www.wordle.net/). Size of text in word clouds is indicative of the number of OTUs within given families.

### Identifying non-functional probe sets

The functionality of each probe set was tested by determining if it would correctly detect the prokMSA reference 16S sequence defined as corresponding to the OTU. We downloaded the PhyloChip taxonomic file from Greengenes, and this file specifies (in most cases) a prokMSA Id for each OTU. These prokMSA Ids were used as a key to extract the corresponding 16S rDNA from the prokMSA reference sequence set (also downloaded from Greengenes). Each of these reference sequences were then turned into a complete set of 25-mers and each of these were matched to the set of probes defined for the corresponding OTU. Those OTUs where every probe did not get matched by at least one 25-mer from the reference were written out for further analysis, and the reasons for the failure determined by examining the probes, the 16S sequence(s) used to derive them and the 16S sequence defined in the prokMSA file.

### Reanalyses of Brodie et al [3]

The abundance data (intensity) for the six largest classes detected in Texas air samples as per Table 1 of Brodie et al. [3], were used to investigate whether probe set results were independent. A random subset, (SA_wk34_ttc, AU_wk19_ttc, AU_wk20_ttc, AU_wk21_ttc, AU_wk22_ttc, AU_wk23_ttc, AU_wk24_ttc, AU_wk25_ttc, AU_wk27_ttc, AU_wk28_ttc, AU_wk29_ttc, AU_wk32_ttc, SA_wk19_ttc, SA_wk20_ttc, SA_wk21_ttc, SA_wk22_ttc, SA_wk23_ttc, SA_wk33_ttc) of Brodie's samples was used . Pearson's correlation coefficients between the abundances of OTUs within each class were calculated in Stata/SE 11.0. Histograms with bin size 0.02 were plotted in SigmaPlot and the counts in each bin scaled to give the same area under the curve. The distribution of Pearson's correlation coefficient expected if the abundances of OTUs were independent of each other was calculated in R using the SuppDists package to find p-values for n equal to 18 then scaling these p-values to give the same area under the curve as the data plots. All scaled counts were plotted in Matlab version 7.7.0(R2008b).

### Reanalysis of OTUs detected by Lemon et al., 2010

OTUs detected by Lemon et al. [20], were obtained from Supplementary Data submitted with their publication. The 1325 detected OTUs were compared with calculated probeset uniqueness for each OTU derived from data downloaded from Greengenes. As Lemon et al [20] used a cutoff of 90%, OTUs with <10% unique probes were counted, and the hypergeometric probability of this many low-uniqueness OTUs being present in a dataset of 1325 OTUs was determined.

## Supporting Information

Methods S1 **Methods for the simulation of the probeset uniqueness data.**
(DOC)

Figure S1 **Probe set uniqueness vs probe set size.** (A) Observed data from the downloaded probe and probe set information from Greengenes. Inset shows number of unique probes, rather than probeset uniqueness. (B) Simulated model of these data. The model does not account for the high-uniqueness, large size probe sets (shown in yellow) or for the number of 0% unique probe sets, which is an order of magnitude greater in data downloaded from Greengenes. Methods for constructing the simulation, and explanatory notes are in Methods S1.
(TIF)

Table S1 **OTUs with no unique probes (left column), OTUs with erroneous probe sets (right column) in the downloaded data set.**
(DOC)

Table S2 **Input taxa for the ISPMA simulation.**
(PDF)

Table S3 **Output taxa for the ISPMA experiment, including number and percentage of matching probes.**
(PDF)

Data S1 **Zip-compressed text file showing OTU numbers, sequences and perfectly matched probes downloaded from Greengenes as directed by Brodie et al** [**17**] **.** OTU numbers were sourced from PhyloTrac and submitted as a query to the tool provided at http://greengenes.lbl.gov/cgi-bin/nph-show_probes_2_otu_alignments.cgi.
(ZIP)
