# Supplementary material for: Reanalysis and Simulation Suggest a Phylogenetic Microarray Does Not Accurately Profile Microbial Communities
Source: PLoS One. 2012 Mar 22;7(3):e33875. doi: 10.1371/journal.pone.0033875 (PMC3310882; doi:10.1371/journal.pone.0033875)
Supplement: Methods S1 — Methods for the simulation of the probeset uniqueness data. (DOC) [file pone.0033875.s001.doc]

Supplementary Material

In order to better understand how probe sets in the ISPMA might have been designed we plotted probe set uniqueness against probe set size (Figure S1A) and performed simulations to try to delineate the different probabilistic characteristics present in these data (Figure S1B). Data from Greengenes and the simulation reveal a general trend where probe set uniqueness declines as probe set size increases. When probe sets are very large, greater than ~ 400 probes, the simulation models which account well for smaller probe sets indicate that the percentage of unique probes per set would be less than 10%. However, some of the large probe sets on the ISPMA are more unique than the simulation suggests and, it would appear, a different process has been applied to these sets. Furthermore, the largest sets are mainly medically important taxa including: *Bacillus anthracis*, *Clostridium botulinum*, *Yersinia pestis* and *Vibrio cholerae*. Presumably, these OTUs have had their probe set sizes increased to attempt to increase accuracy. Finally, the number of probe sets on the PhyloChip that have no unique probes were greater by an order of magnitude than the simulation models would predict, hence these constitute another probabilistic class.

As depicted in Figure S1b, the essential features of the uniqueness – probeset size relationship can be accounted for by a simple simulation consisting of two components. In both models, a probability of uniqueness is assigned to each probe which decreases with the order of probes. In the high-probability model, the first 26 probes have a uniqueness probability of 4/6, which decreases to zero in stages by the 40^th^ probe (3/5 for probes 27-32; 1/2 for probes 33-35; 1/3 for probes 36-39). In the low-probability model, the probabilities are 15/100 for probes 1-30, 10/100 for probes 31-60, 5/100 for probes 61-90, and zero thereafter. The lower-probability model was applied to the 2400 probesets with the lowest uniqueness in the actual data. The higher-probability model was applied to the remaining 6535 probesets. For each probe in a given probeset, the corresponding model generates a random number between 0 and 1, and if this number is less than (or equal to) the attributed probability, it deems the probe unique.

The simulation matches the mean uniqueness of the actual data (and hence the total number of unique probes) and the power law which forms the upper limit of uniqueness, max(uniqueness) ≈28/probeset size. The simulation also matches the microcosmic features of the data, where there is localised convergence towards a particular ratio (e.g. .5, .66, etc.).

What the simulation does not account for is just as useful. Firstly, there are the outlying probesets with uniqueness which exceeds the power law (28/probeset size). Of note, are the probesets of size >400 (with unique probes far in excess of 28) many of which appear to be medically relevant. Second, if we include the OTUs with fewer than 11 probes, there are 2047 actual probesets with not a single unique probe.

In summary, then, there are at least four different probabilistic classes of probesets on the microarray – the two classes represented by the higher-probability and the lower-probability models in the simulation, the outliers with uniqueness that exceeds 28/probeset size, and finally, the class of probe sets that contains entirely non-unique probes.
