# Supplementary figures and images for: Reanalysis and Simulation Suggest a Phylogenetic Microarray Does Not Accurately Profile Microbial Communities
Source: PLoS One. 2012 Mar 22;7(3):e33875. doi: 10.1371/journal.pone.0033875 (PMC3310882; doi:10.1371/journal.pone.0033875)

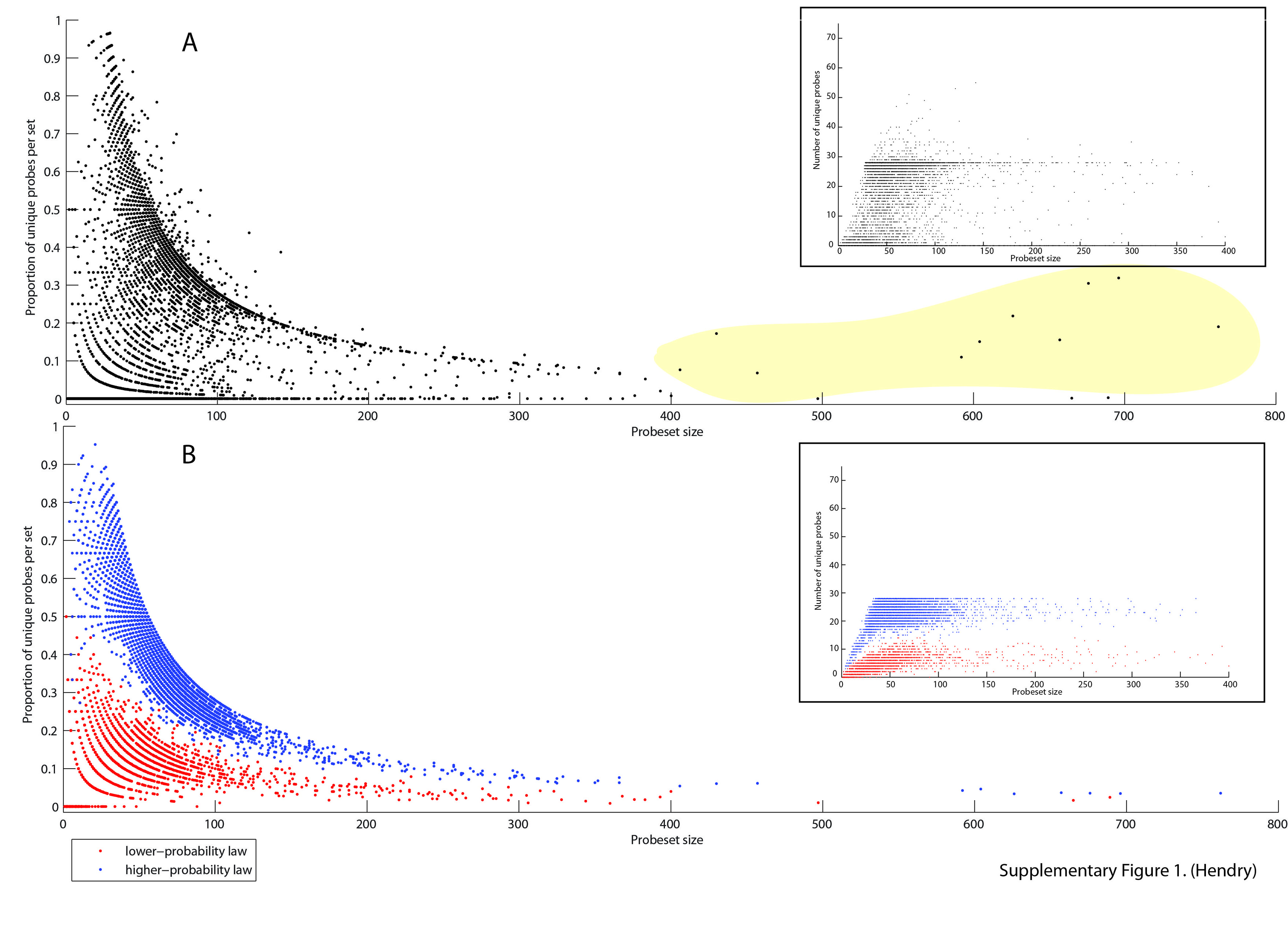

Supplement: Figure S1 — Probe set uniqueness vs probe set size. (A) Observed data from the downloaded probe and probe set information from Greengenes. Inset shows number of unique probes, rather than probeset uniqueness. (B) Simulated model of these data. The model does not account for the high-uniqueness, large size probe sets (shown in yellow) or for the number of 0% unique probe sets, which is an order of magnitude greater in data downloaded from Greengenes. Methods for constructing the simulation, and explanatory notes are in Methods S1. (TIF) [file pone.0033875.s002.tif]
