# Supplementary material for: Reanalysis and Simulation Suggest a Phylogenetic Microarray Does Not Accurately Profile Microbial Communities
Source: PLoS One. 2012 Mar 22;7(3):e33875. doi: 10.1371/journal.pone.0033875 (PMC3310882; doi:10.1371/journal.pone.0033875)
Supplement: Table S1 — OTUs with no unique probes (left column), OTUs with erroneous probe sets (right column) in the downloaded data set. (DOC) [file pone.0033875.s003.doc]

Supplementary Table 1.

| OTU probe sets with no unique probes | OTUs with erroneous probe sets |
| --- | --- |
| 11, 13, 17, 21, 27, 28, 38, 40, 51, 53, 58, 60, 61, 66, 71, 76, 77, 87, 89, 91, 94, 95, 100, 105, 106, 117, 118, 119, 131, 132, 140, 144, 149, 155, 169, 176, 179, 181, 196, 197, 198, 203, 205, 206, 208, 212, 224, 226, 227, 234, 239, 242, 244, 246, 249, 252, 258, 262, 268, 269, 270, 272, 283, 287, 288, 300, 304, 305, 312, 319, 326, 327, 333, 344, 346, 349, 350, 353, 355, 366, 369, 374, 375, 385, 393, 419, 428, 429, 433, 437, 452, 462, 468, 482, 484, 485, 490, 508, 514, 515, 522, 528, 533, 534, 536, 541, 542, 544, 546, 547, 548, 549, 556, 557, 558, 559, 560, 562, 563, 569, 571, 572, 576, 582, 583, 594, 601, 613, 616, 625, 628, 629, 636, 637, 642, 644, 646, 651, 652, 655, 661, 663, 671, 676, 681, 683, 686, 687, 691, 697, 699, 701, 705, 706, 709, 710, 712, 719, 721, 722, 726, 734, 737, 740, 747, 751, 757, 760, 766, 767, 768, 769, 789, 790, 792, 806, 816, 818, 819, 829, 832, 861, 863, 884, 885, 895, 897, 920, 926, 927, 946, 964, 969, 984, 987, 993, 994, 1015, 1034, 1041, 1046, 1048, 1049, 1050, 1051, 1071, 1087, 1089, 1090, 1104, 1105, 1109, 1111, 1115, 1116, 1119, 1120, 1135, 1146, 1149, 1158, 1159, 1161, 1175, 1177, 1180, 1184, 1186, 1187, 1190, 1191, 1197, 1201, 1204, 1211, 1213, 1224, 1238, 1239, 1240, 1241, 1261, 1262, 1266, 1274, 1282, 1286, 1296, 1304, 1308, 1313, 1317, 1324, 1340, 1343, 1348, 1350, 1351, 1354, 1355, 1360, 1365, 1375, 1385, 1392, 1403, 1405, 1428, 1429, 1431, 1432, 1435, 1436, 1446, 1449, 1450, 1454, 1461, 1463, 1488, 1492, 1494, 1504, 1517, 1542, 1544, 1545, 1546, 1552, 1557, 1565, 1567, 1569, 1573, 1576, 1581, 1582, 1583, 1585, 1586, 1587, 1589, 1593, 1598, 1600, 1610, 1611, 1612, 1615, 1617, 1633, 1637, 1639, 1644, 1650, 1652, 1654, 1667, 1669, 1671, 1676, 1677, 1681, 1684, 1686, 1687, 1690, 1692, 1695, 1696, 1711, 1726, 1732, 1741, 1743, 1745, 1748, 1749, 1758, 1760, 1766, 1769, 1770, 1771, 1779, 1780, 1781, 1786, 1787, 1790, 1791, 1801, 1803, 1804, 1805, 1820, 1822, 1824, 1834, 1835, 1843, 1852, 1854, 1863, 1867, 1872, 1875, 1885, 1888, 1889, 1910, 1917, 1921, 1923, 1927, 1931, 1940, 1961, 1966, 1967, 1970, 1972, 1983, 1990, 2005, 2014, 2031, 2034, 2040, 2047, 2048, 2049, 2051, 2052, 2053, 2086, 2088, 2100, 2101, 2110, 2127, 2129, 2130, 2136, 2137, 2143, 2144, 2147, 2148, 2149, 2153, 2165, 2195, 2213, 2214, 2215, 2223, 2224, 2237, 2238, 2240, 2241, 2242, 2254, 2264, 2272, 2285, 2324, 2338, 2339, 2359, 2364, 2367, 2380, 2385, 2388, 2397, 2398, 2432, 2433, 2436, 2438, 2443, 2445, 2456, 2485, 2487, 2488, 2489, 2490, 2491, 2493, 2497, 2523, 2537, 2541, 2545, 2554, 2558, 2559, 2582, 2583, 2585, 2586, 2587, 2588, 2589, 2591, 2593, 2594, 2621, 2625, 2664, 2668, 2679, 2683, 2694, 2698, 2701, 2710, 2714, 2716, 2721, 2729, 2736, 2754, 2793, 2794, 2797, 2804, 2805, 2816, 2825, 2834, 2844, 2849, 2900, 2913, 2915, 2917, 2921, 2923, 2931, 2937, 2946, 2961, 2988, 2991, 2993, 2994, 3017, 3019, 3021, 3025, 3030, 3036, 3044, 3049, 3059, 3060, 3075, 3076, 3077, 3080, 3084, 3087, 3089, 3108, 3111, 3112, 3171, 3182, 3217, 3250, 3251, 3253, 3258, 3261, 3265, 3266, 3279, 3283, 3284, 3285, 3287, 3289, 3290, 3298, 3299, 3301, 3313, 3323, 3326, 3328, 3330, 3337, 3342, 3344, 3345, 3362, 3365, 3366, 3368, 3382, 3385, 3392, 3397, 3398, 3401, 3415, 3419, 3422, 3424, 3430, 3433, 3434, 3437, 3439, 3441, 3446, 3460, 3467, 3476, 3478, 3482, 3488, 3489, 3490, 3491, 3492, 3497, 3499, 3500, 3504, 3508, 3514, 3517, 3522, 3540, 3545, 3552, 3555, 3559, 3561, 3566, 3569, 3579, 3583, 3588, 3589, 3598, 3605, 3612, 3617, 3627, 3628, 3629, 3632, 3633, 3634, 3635, 3638, 3641, 3651, 3654, 3659, 3661, 3675, 3679, 3684, 3685, 3687, 3688, 3706, 3713, 3725, 3730, 3745, 3753, 3756, 3758, 3763, 3767, 3768, 3769, 3773, 3780, 3794, 3802, 3818, 3822, 3827, 3831, 3833, 3836, 3840, 3842, 3843, 3845, 3860, 3865, 3869, 3873, 3879, 3881, 3887, 3894, 3900, 3901, 3904, 3906, 3909, 3917, 3918, 3922, 3925, 3929, 3937, 3951, 3955, 3956, 3961, 3965, 3972, 3975, 3976, 3981, 3993, 4012, 4044, 4045, 4046, 4047, 4102, 4112, 4154, 4155, 4156, 4177, 4180, 4185, 4187, 4212, 4214, 4222, 4225, 4229, 4244, 4261, 4267, 4272, 4275, 4278, 4280, 4281, 4286, 4289, 4293, 4297, 4298, 4299, 4300, 4306, 4321, 4325, 4326, 4330, 4331, 4335, 4339, 4341, 4357, 4359, 4364, 4369, 4377, 4405, 4406, 4415, 4418, 4427, 4434, 4459, 4461, 4477, 4480, 4497, 4503, 4507, 4510, 4511, 4512, 4514, 4524, 4525, 4526, 4533, 4535, 4536, 4538, 4539, 4550, 4554, 4555, 4559, 4560, 4566, 4567, 4571, 4572, 4575, 4582, 4584, 4587, 4589, 4590, 4598, 4607, 4613, 4614, 4616, 4622, 4637, 4652, 4668, 4670, 4677, 4683, 4685, 4694, 4698, 4702, 4704, 4709, 4720, 4740, 4749, 4801, 4802, 4818, 4855, 4895, 4897, 4900, 4912, 4913, 4931, 4948, 4966, 4970, 4976, 4978, 4983, 4984, 4993, 5000, 5001, 5004, 5007, 5008, 5010, 5015, 5017, 5022, 5027, 5028, 5030, 5032, 5034, 5038, 5039, 5040, 5047, 5049, 5051, 5057, 5060, 5061, 5064, 5072, 5077, 5078, 5111, 5115, 5130, 5131, 5156, 5164, 5166, 5174, 5175, 5176, 5177, 5182, 5183, 5184, 5189, 5190, 5191, 5199, 5210, 5215, 5219, 5235, 5248, 5257, 5267, 5270, 5294, 5295, 5301, 5307, 5317, 5322, 5334, 5352, 5353, 5355, 5366, 5368, 5372, 5389, 5398, 5400, 5401, 5416, 5434, 5435, 5436, 5439, 5454, 5456, 5457, 5462, 5463, 5474, 5475, 5477, 5479, 5480, 5482, 5484, 5492, 5495, 5499, 5500, 5510, 5521, 5542, 5544, 5545, 5547, 5563, 5567, 5573, 5605, 5619, 5651, 5652, 5663, 5664, 5668, 5680, 5695, 5714, 5716, 5728, 5783, 5793, 5799, 5802, 5817, 5820, 5822, 5832, 5843, 5874, 5875, 5888, 5889, 5890, 5891, 5892, 5896, 5901, 5902, 5903, 5904, 5905, 5913, 5914, 5916, 5919, 5926, 5928, 5940, 5944, 5945, 5947, 5951, 5954, 5955, 5956, 5958, 5971, 5991, 5992, 5994, 5996, 5997, 5998, 6011, 6012, 6019, 6030, 6044, 6050, 6053, 6066, 6069, 6083, 6084, 6090, 6091, 6123, 6124, 6143, 6146, 6158, 6185, 6208, 6212, 6244, 6249, 6250, 6252, 6261, 6267, 6271, 6298, 6317, 6319, 6324, 6335, 6337, 6339, 6345, 6348, 6349, 6350, 6353, 6355, 6356, 6357, 6359, 6360, 6362, 6363, 6364, 6366, 6367, 6368, 6408, 6410, 6414, 6415, 6421, 6422, 6423, 6424, 6426, 6430, 6458, 6459, 6460, 6463, 6466, 6468, 6469, 6470, 6476, 6477, 6480, 6488, 6489, 6490, 6491, 6492, 6496, 6503, 6506, 6507, 6508, 6512, 6513, 6514, 6523, 6526, 6532, 6535, 6547, 6549, 6552, 6554, 6557, 6562, 6565, 6567, 6568, 6571, 6573, 6576, 6579, 6636, 6638, 6639, 6648, 6650, 6651, 6652, 6653, 6660, 6661, 6662, 6663, 6665, 6670, 6679, 6683, 6690, 6692, 6694, 6697, 6699, 6700, 6701, 6714, 6732, 6735, 6737, 6738, 6740, 6750, 6757, 6758, 6761, 6762, 6766, 6768, 6770, 6781, 6783, 6790, 6798, 6799, 6803, 6804, 6805, 6809, 6810, 6821, 6822, 6823, 6830, 6836, 6857, 6862, 6867, 6871, 6878, 6883, 6887, 6888, 6890, 6895, 6899, 6904, 6908, 6909, 6916, 6917, 6918, 6922, 6923, 6926, 6927, 6928, 6941, 6945, 6947, 6954, 6955, 6959, 6960, 6962, 6964, 6966, 6968, 6971, 6972, 6975, 6980, 6981, 6986, 6991, 6992, 6995, 7007, 7008, 7009, 7010, 7025, 7026, 7027, 7028, 7029, 7035, 7036, 7038, 7040, 7041, 7043, 7044, 7051, 7056, 7060, 7067, 7072, 7075, 7076, 7084, 7085, 7087, 7091, 7100, 7101, 7102, 7109, 7110, 7115, 7123, 7125, 7126, 7135, 7137, 7139, 7141, 7147, 7150, 7153, 7155, 7156, 7157, 7165, 7182, 7188, 7194, 7216, 7222, 7226, 7232, 7257, 7263, 7264, 7275, 7284, 7285, 7299, 7300, 7308, 7310, 7316, 7323, 7333, 7334, 7336, 7341, 7352, 7355, 7359, 7362, 7364, 7366, 7373, 7374, 7377, 7379, 7381, 7383, 7384, 7385, 7390, 7394, 7398, 7400, 7401, 7403, 7405, 7415, 7419, 7431, 7433, 7436, 7437, 7439, 7440, 7445, 7453, 7456, 7459, 7471, 7477, 7481, 7482, 7493, 7497, 7508, 7511, 7522, 7528, 7529, 7535, 7537, 7543, 7545, 7548, 7550, 7556, 7560, 7568, 7572, 7575, 7576, 7584, 7591, 7596, 7597, 7600, 7606, 7634, 7640, 7649, 7674, 7682, 7704, 7705, 7718, 7719, 7737, 7743, 7745, 7751, 7761, 7762, 7764, 7765, 7770, 7771, 7778, 7782, 7784, 7788, 7789, 7796, 7800, 7805, 7807, 7808, 7809, 7817, 7822, 7823, 7824, 7829, 7837, 7838, 7843, 7845, 7847, 7856, 7857, 7858, 7859, 7865, 7866, 7870, 7871, 7878, 7888, 7892, 7893, 7902, 7907, 7910, 7917, 7919, 7921, 7925, 7928, 7931, 7932, 7935, 7941, 7942, 7945, 7948, 7949, 7957, 7959, 7960, 7964, 7965, 7968, 7969, 7971, 7975, 7976, 7978, 7983, 7984, 7985, 7986, 7987, 7990, 7992, 8001, 8003, 8009, 8011, 8012, 8013, 8018, 8021, 8023, 8031, 8032, 8034, 8037, 8041, 8044, 8045, 8046, | 3, 16, 23, 25, 52, 85, 163, 190, 212, 270, 282, 285, 298, 312, 313, 332, 336, 364, 377, 404, 408, 409, 489, 492, 500, 512, 529, 537, 653, 657, 667, 693, 717, 764, 770, 826, 891, 907, 909, 919, 953, 958, 960, 966, 968, 969, 999, 1007, 1026, 1051, 1054, 1119, 1122, 1178, 1193, 1206, 1212, 1221, 1232, 1246, 1300, 1334, 1345, 1347, 1352, 1395, 1414, 1416, 1444, 1445, 1447, 1470, 1472, 1482, 1495, 1496, 1546, 1555, 1570, 1575, 1591, 1636, 1643, 1668, 1671, 1672, 1698, 1714, 1746, 1757, 1839, 1846, 1883, 1910, 1915, 1928, 1930, 1946, 1953, 1991, 1999, 2009, 2019, 2028, 2034, 2057, 2059, 2129, 2135, 2175, 2190, 2191, 2200, 2201, 2213, 2237, 2269, 2270, 2304, 2338, 2342, 2351, 2355, 2362, 2366, 2370, 2422, 2441, 2451, 2459, 2471, 2495, 2510, 2530, 2546, 2555, 2576, 2585, 2596, 2725, 2735, 2804, 2862, 2883, 2892, 2906, 2914, 2974, 2981, 2984, 3016, 3028, 3049, 3066, 3108, 3112, 3126, 3137, 3149, 3173, 3179, 3189, 3202, 3213, 3236, 3249, 3252, 3278, 3282, 3293, 3302, 3311, 3324, 3349, 3352, 3363, 3364, 3379, 3391, 3400, 3416, 3455, 3475, 3492, 3502, 3505, 3515, 3519, 3540, 3567, 3568, 3572, 3583, 3614, 3619, 3628, 3631, 3665, 3684, 3715, 3753, 3779, 3797, 3806, 3809, 3810, 3821, 3862, 3864, 3866, 3868, 3871, 3881, 3885, 3886, 3893, 3907, 3913, 3923, 3931, 3960, 3971, 3973, 3985, 4004, 4040, 4041, 4045, 4053, 4062, 4065, 4083, 4088, 4096, 4117, 4120, 4134, 4159, 4183, 4193, 4309, 4324, 4371, 4389, 4391, 4440, 4442, 4445, 4534, 4544, 4551, 4560, 4568, 4576, 4581, 4583, 4585, 4588, 4590, 4712, 4736, 4750, 4753, 4767, 4773, 4792, 4796, 4810, 4814, 4821, 4828, 4837, 4883, 4884, 4888, 4899, 4911, 4916, 4927, 4942, 4943, 4958, 4987, 4990, 4994, 5072, 5095, 5106, 5122, 5145, 5197, 5198, 5240, 5249, 5317, 5320, 5321, 5365, 5374, 5420, 5421, 5435, 5459, 5462, 5467, 5526, 5551, 5630, 5640, 5648, 5686, 5713, 5746, 5785, 5806, 5836, 5884, 5921, 5997, 6000, 6040, 6072, 6075, 6125, 6166, 6187, 6198, 6214, 6325, 6364, 6383, 6384, 6518, 6539, 6548, 6561, 6585, 6604, 6612, 6615, 6616, 6622, 6634, 6675, 6703, 6724, 6758, 6772, 6857, 6878, 6886, 6912, 6917, 6937, 6945, 6980, 7026, 7032, 7068, 7105, 7106, 7116, 7118, 7234, 7269, 7290, 7321, 7336, 7349, 7398, 7404, 7464, 7469, 7483, 7507, 7568, 7583, 7606, 7615, 7640, 7651, 7665, 7666, 7695, 7700, 7701, 7712, 7753, 7807, 7809, 7822, 7834, 7860, 7880, 7887, 7897, 7898, 7909, 7913, 7922, 7929, 7934, 7947, 7970, 7986, 8052, 8067, 8076, 8097, 8102, 8139, 8178, 8183, 8195, 8220, 8221, 8259, 8273, 8309, 8344, 8374, 8388, 8406, 8408, 8434, 8440, 8455, 8456, 8485, 8499, 8505, 8508, 8510, 8536, 8559, 8564, 8580, 8583, 8626, 8629, 8630, 8632, 8633, 8649, 8673, 8693, 8715, 8716, 8771, 8772, 8783, 8800, 8804, 8819, 8838, 8844, 8856, 8859, 8942, 8954, 8958, 8995, 9000, 9056, 9078, 9082, 9099, 9107, 9112, 9117, 9136, 9140, 9148, 9168, 9202, 9204, 9211, 9222, 9225, 9247, 9251, 9259, 9274, 9298, 9358, 9374, 9400, 9402, 9430, 9442, 9443, 9526, 9530, 9542, 9546, 9612, 9617, 9646, 9660, 9680, 9713, 9789, 9842, 9844, 9886, 9982, 9983, 10046, 10065, 10125, 10149, 10186, 10201, 10263, 10288, 10300, 10306, 10314, 10318, 10358, 10364, 10367, 10416, 10420, 10427, 10451, 10456, 10460, 10525, 10541, 10548, 10566, 10578, |
