# Supplementary material for: Reanalysis and Simulation Suggest a Phylogenetic Microarray Does Not Accurately Profile Microbial Communities
Source: PLoS One. 2012 Mar 22;7(3):e33875. doi: 10.1371/journal.pone.0033875 (PMC3310882; doi:10.1371/journal.pone.0033875)
Supplement: Table S2 — Input taxa for the ISPMA simulation. (PDF) [file pone.0033875.s004.pdf]

Supplementary Table 2. Input taxa for the ISPMA experiment. RDP Accession code and description of taxon.

>S000000041 Ethiopian Gliricidia little leaf phytoplasma; GLL-Eth; AF361018	Acholeplasmataceae
>S000003595 uncultured eubacterium; 611; Y11629	Acidobacteria_Gp1
>S000345971 uncultured archaeon; GBa1r015; AF419628	Archaeoglobaceae
>S000006374 Alkalibacillus haloalkaliphilus (T); DSM 5271T; AJ238041	Bacillaceae
>S000414380 Nitrobacter winogradskyi; W; L11661	Bradyrhizobiaceae
>S000000049 Burkholderia sp. S-2; AB079372	Burkholderiaceae
>S000004159 Campylobacter lanienae; FK172; AB076676	Campylobacteraceae
>S000014722 Chlorobium limicola; M1; AB054671	Chlorobiaceae
>S000000168 Clostridium beijerinckii; JCM 7833; AB020187	Clostridiaceae 1
>S000319462 Finegoldia magna; ATCC 29328; AB109769	Clostridiales XI
>S000387151 Acidaminobacter hydrogenoformans (T); glu 65; AF016691	Clostridiales XII
>S000108680 uncultured Gram-positive bacterium; ikaite un-c18; AJ431343	Clostridiales XIV
>S000001712 Acidovorax sp. KSP2; AB076843	Comamonadaceae
>S000428537 Denitrobacterium detoxificans; NPOH2; AF079505	Coriobacteriaceae
>S000562034 uncultured actinobacterium; APe2_64; AB074644	Dietziaceae
>S000389369 Flexistipes sp. vp180; AF220344	Deferribacteraceae
>S000088673 Desulfobacterium indolicum (T); DSM 3383; marine mud; AJ237607	Desulfobacteraceae
>S000010890 sulfate-reducing bacterium R-PropA1; AJ012591	Desulfobulbaceae
>S001548750 Desulfovibrio magneticus RS-1; AP010904	Desulfovibrionaceae
>S000381431 uncultured crenarchaeote; CB9; X99563	Desulfurococcaceae
>S000379948 uncultured Desulfuromonas sp.; M76; AY692042	Desulfuromonadaceae
>S000857281 uncultured bacterium; 16saw44-1g06.p1ka; EF604201	Enterobacteriaceae
>S000330866 Alkalibacterium sp. 12A2; AY554413	Eubacteriaceae
>S000000315 Halomonas subglaciescola (T); DSM 4683; AJ306892	Halomonadaceae
>S000126553 uncultured bacterium; sipK119; AJ307940	Helicobacteraceae
>S000118790 uncultured beta proteobacterium; ST01-SN1D; AY222297	Hydrogenophilaceae
>S000000130 Kineococcus-like str. AS3642; AF095334	Kineosporiaceae
>S000273566 Anaerosporobacter mobilis (T); IMSNU 40011; AY534872	Lachnospiraceae
>S000002359 Listeria ivanovii (T); CLIP12229; X98529	Listeriaceae
>S000022636 Anaerophaga thermohalophila (T); Fru22; AJ418048	Marinilabiaceae
>S000414785 Methanobacterium subterraneum (T); A8p, DSM 11074; X99044	Methanobacteriaceae
>S000342897 uncultured archaeon; G26_C49; AF356632	Methanococcaceae
>S000345977 uncultured archaeon; C1_R002; AF419634	Methanocorpusculaceae
>S000379966 uncultured Methanospirillum sp.; M40; AY692060	Methanospirillaceae
>S000336840 uncultured archaeon WCHD3-03; AF050611	Methanosaetaceae
>S000000727 Methylocystis sp. H9a; AJ458490	Methylocystaceae
>S000000100 Cellulomonas sp.; 794; Y09656	Cellulomonadaceae
>S000001583 Desulfitobacterium hafniense (T); DCB-2; X94975	Peptococcaceae 1
>S000147781 uncultured Bacteroidetes bacterium; S15B-MN34; AJ583190	Porphyromonadaceae
>S000008544 Propionibacterium propionicum (T); DSM 43307; AJ003058	Propionibacteriaceae
>S000002510 Pseudomonas stutzeri; 24a13; soil contaminated with mineral oil; AJ270451	Pseudomonadaceae
>S000381427 Pyrodictium abyssi; DSM 6158; X99559	Pyrodictiaceae
>S000000452 Rhizobium genosp. R; BDV5365; Z94803	Rhizobiaceae
>S000122843 Pannonibacter phragmitetus; C6/8; AJ314748	Rhodobacteraceae
>S000000311 Azoarcus anaerobius (T); LuFres1; DSM 12081; Y14701	Rhodocyclaceae
>S000000835 Sporobacter termitidis (T); SYR; Z49863	Ruminococcaceae
>S000000164 Shewanella sp.; 16.k; NB1-a; AB013822	Shewanellaceae
>S000001467 Sphingomonas echinoides (T); ATCC 14820T; AB021370	Sphingomonadaceae
>S000125645 uncultured bacterium; B01R001; AY197374	Spirochaetaceae
>S000018874 Anaerobaculum mobile (T); type strain: NGA = DSM13181 = ATCC BAA-54; AJ243189	Synergistaceae
>S000005091 Clostridium uzonii; DSM 9752; Y18182	Thermoanaerobacteraceae
>S000391948 Thermoplasma volcanium; GSS1; DSM 4299; AF339746	Thermoplasmataceae
>S000002370 magnetic coccus MP17; X80996	Rhodospirillaceae
>S000821835 uncultured rumen bacterium; BRC30; EF436316	Prevotellaceae
>S000130406 uncultured rape rhizosphere bacterium wr0025; AJ295484	Rhodocyclaceae
>S000003496 Dehalobacterium formicoaceticum; DMC; X86690	Veillonellaceae
>S000020221 uncultured delta proteobacterium Sva0485; AJ241001	Desulfarculaceae
>S000354993 uncultured epsilon proteobacterium; 44a-B1-40; AY082468	Helicobacteraceae
>S000001464 Eubacterium cylindroides; JCM 7787; AB018187	Eubacteriaceae
>S000000039 Cytophaga sp. 41-DBG2; AF427479	Flavobacteriaceae
>S000016974 uncultured gamma proteobacterium Sva1046; AJ240991	Sedimenticola
>S001558978 uncultured Anaeroglobus sp.; 10B655; FJ976316	Veillonellaceae
>S000018585 uncultured verrucomicrobium DEV009; AJ401108	Verrucomicrobiaceae
>S000009721 Vibrio ordalii (T); ATCC 33509T; X74718	Vibrionaceae
