# Supplementary material for: Reanalysis and Simulation Suggest a Phylogenetic Microarray Does Not Accurately Profile Microbial Communities
Source: PLoS One. 2012 Mar 22;7(3):e33875. doi: 10.1371/journal.pone.0033875 (PMC3310882; doi:10.1371/journal.pone.0033875)
Supplement: Table S3 — Output taxa for the ISPMA experiment, including number and percentage of matching probes. (PDF) [file pone.0033875.s005.pdf]

Supplementary Table 3. Output taxa for the ISPMA experiment

Accession number	Domain	Phylum	RDP Family	*Genus* /Subfamily	Description	OTU#	probes	matches	% of probes that match
AF361018.1	Bacteria	Tenericutes	Acholeplasmataceae	*Acholeplasma*	Ethiopia isolate str. GLL-Eth	4138	62	62	100.00%
AF407383.1	Bacteria	Proteobacteria	Beijerinckiaceae	*Methylovirgula*	Methylocella tundrae str. Y1	7153	68	68	100.00%
Z94814.1	Bacteria	Proteobacteria	Bradyrhizobiaceae	*Bradyrhizobium*	Bradyrhizobium japonicum	6917	34	34	100.00%
D12781.1	Bacteria	Proteobacteria	Bradyrhizobiaceae	*Bradyrhizobium*	Bradyrhizobium japonicum SD5	6878	33	33	100.00%
M65248.1	Bacteria	Proteobacteria	Bradyrhizobiaceae	*Afipia*	Oligotropha carboxidovorans str. S23	7403	33	33	100.00%
L35506.1	Bacteria	Proteobacteria	Bradyrhizobiaceae	*Nitrobacter*	Nitrobacter hamburgensis	6927	26	26	100.00%
L11664.1	Bacteria	Proteobacteria	Bradyrhizobiaceae	*Bradyrhizobium*	Rhodopseudomonas rhenobacensis str. Klemme Rb	6941	16	16	100.00%
AF078758.1	Bacteria	Proteobacteria	Burkholderiales_incertae_sedis	*Xylophilus*	Xylophilus ampelinus str. ATCC 33914	7829	30	30	100.00%
AF043423.1	Bacteria	Proteobacteria	Campylobacteraceae	*Campylobacter*	Campylobacter lanienae str. FK176	10553	39	39	100.00%
L04319.1	Bacteria	Proteobacteria	Campylobacteraceae	*Campylobacter*	Campylobacter showae	10456	19	19	100.00%
X79459.1	Bacteria	Actinobacteria	Cellulomonadaceae	Micrococcineae	Cellulomonas gelida	1586	27	27	100.00%
AB054671.1	Bacteria	Chlorobi	Chlorobiaceae	*Chlorobium*	Chlorobaculum sp.M1	995	50	50	100.00%
Y08108.1	Bacteria	Chlorobi	Chlorobiaceae	*Chlorobium*	Chlorobium ferrooxidans	262	19	19	100.00%
X77843.1	Bacteria	Firmicutes	Clostridiaceae 1	Clostridiaceae 1	Clostridium beijerinckii	4560	243	243	100.00%
AY169413.1	Bacteria	Firmicutes	Clostridiales XI	*Finegoldia*	Finegoldia magna str. ATCC 29328	224	37	37	100.00%
AF016691.1	Bacteria	Firmicutes	Clostridiales XII	*Acidaminobacter*	Acidaminobacter hydrogenoformans	2990	103	103	100.00%
AF078760.1	Bacteria	Proteobacteria	Comamonadaceae	*Acidovorax*	Acidovorax konjaci str. DSM 7481	8012	51	51	100.00%
AF078771.1	Bacteria	Proteobacteria	Comamonadaceae	*Acidovorax*	Hydrogenophaga flava str. DSM 619T	7888	27	27	100.00%
AJ224987.1	Bacteria	Proteobacteria	Comamonadaceae	*Polaromonas*	Polaromonas naphthalenivorans str. CJ2	7822	26	26	100.00%
AF079505.1	Bacteria	Actinobacteria	Coriobacteriaceae	Coriobacterineae	Denitrobacterium detoxificans str. MAJ1	1145	62	62	100.00%
AY274839.1	Bacteria	Bacteroidetes	Cryomorphaceae	*Owenweeksia*	Delaware River estuary clone 1G12	5248	15	15	100.00%
AJ012591.1	Bacteria	Proteobacteria	Desulfobulbaceae	*Desulfobulbus*	related to Desulfobulbus	9891	63	63	100.00%
AJ240991.1	Bacteria	Proteobacteria	Ectothiorhodospiraceae	*Alkalispirillum*	Norway:(Svalbard)Hornsund clone Sva1046	9387	84	84	100.00%
AF075271.2	Bacteria	Proteobacteria	Enterobacteriaceae	*Escherichia/Shigella*	Alterococcus agarolyticus	8783	157	157	100.00%
U18997.1	Bacteria	Proteobacteria	Enterobacteriaceae	*Escherichia/Shigella*	Salmonella subsp. enterica serovar Waycross str. Swy1 subsp.	9358	93	93	100.00%
U90318.1	Bacteria	Proteobacteria	Enterobacteriaceae	*Serratia*		8640	24	24	100.00%
AB018187.1	Bacteria	Firmicutes	Eubacteriaceae	*Eubacterium*	cylindroides str. JCM 7787	4013	64	64	100.00%
AF427479.1	Bacteria	Bacteroidetes	Flavobacteriaceae	*Krokinobacter*	Cytophaga sp. str. 41-DBG2	5697	71	71	100.00%
AJ306892.1	Bacteria	Proteobacteria	Halomonadaceae	*Halomonas*	Halomonas subglaciescola str. DSM 4683	8242	80	80	100.00%
AB053951.1	Bacteria	Proteobacteria	Helicobacteraceae	*Sulfuricurvum*	Sulfuricurvum kujiense str. YK-3	10550	41	41	100.00%
AY222297.1	Bacteria	Proteobacteria	Hydrogenophilaceae	*Hydrogenophilus*	silica sinter depositing geothermal power station discharge drain clone ST01-SN1D proteobacterium	7975	88	88	100.00%
AF165267.1	Bacteria	Proteobacteria	Hyphomicrobiaceae	*Rhodoplanes*	Beijerinckia indica	6651	18	18	100.00%
X77958.1	Bacteria	Actinobacteria	Kineosporiaceae	Kineosporiineae	Kineococcus aurantiacus	1961	22	22	100.00%
X98529.1	Bacteria	Firmicutes	Listeriaceae	*Listeria*	Listeria innocua Clip11262	3701	60	60	100.00%
AJ441239.1	Bacteria	Bacteroidetes	Marinilabiaceae	*Alkaliflexus*	Paralvinella palmiformis mucus secretions clone P. palm 53 bacterium	5874	11	11	100.00%
U55233.1	Archaea	Euryarchaeota	Methanobacteriaceae	*Methanobrevibacter*	Methanobrevibacter smithii	2241	18	18	100.00%
AF054208.1	Archaea	Euryarchaeota	Methanobacteriaceae	*Methanobrevibacter*	Methanobrevibacter smithii	2110	15	15	100.00%
AF356632.1	Archaea	Euryarchaeota	Methanococcaceae	*Methanothermococcus*	Guaymas Basin clone G26_C49	2147	35	35	100.00%
AF095268.1	Archaea	Euryarchaeota	Methanocorpusculaceae	*Methanocorpusculum*	Methanocorpusculum parvum str. DSM 3828	2278	34	34	100.00%
AF050611.1	Archaea	Euryarchaeota	Methanosaetaceae	*Methanosaeta*	contaminated aquifer clone WCHD3-03	2130	77	77	100.00%
AF423188.1	Archaea	Euryarchaeota	Methanosaetaceae	*Methanosaeta*	biodegraded Canadian oil reservoir clone PL-35A3	2153	77	77	100.00%
AJ009508.1	Archaea	Euryarchaeota	Methanosaetaceae	*Methanosaeta*	UASB reactor clone M6	2129	59	59	100.00%
L20845.1	Bacteria	Proteobacteria	Methylocystaceae	*Methylocystis*	Methylosinus trichosporium	7640	39	39	100.00%
X86601.1	Bacteria	Actinobacteria	Microbacteriaceae	Micrococcineae	Jonesia quinghaiensis str. DSM 15701	1687	12	12	100.00%
U12224.1	Bacteria	Actinobacteria	Micrococcaceae	Micrococcineae	Arthrobacter ureafaciens	1405	13	13	100.00%
U68528.1	Bacteria	Firmicutes	Peptococcaceae 1	Peptococcaceae 1	Desulfitobacterium hafniense str. DCB-2	832	55	55	100.00%
U40078.1	Bacteria	Firmicutes	Peptococcaceae 1	Peptococcaceae 1	Desulfitobacterium frappieri	816	54	54	100.00%
X82823.1	Bacteria	Bacteroidetes	Porphyromonadaceae	*Porphyromonas*	Porphyromonas catoniae	5713	28	28	100.00%
AJ318126.1	Bacteria	Bacteroidetes	Porphyromonadaceae	*Dysgonomonas*	Dysgonomonas wimpennyi str. ANFA2	5454	14	14	100.00%
AY218551.1	Bacteria	Bacteroidetes	Porphyromonadaceae	*Proteiniphilum*	penguin droppings sediments clone KD1-125	5890	13	13	100.00%
X83808.1	Bacteria	Actinobacteria	Promicromonosporaceae	*Micrococcineae*	Beutenbergia cavernosa str. DSM 12333	1748	15	15	100.00%
Z94803.1	Bacteria	Proteobacteria	Rhizobiaceae	*Rhizobium*	Rhizobium genosp. R str. BDV5365	6813	201	201	100.00%
U28916.1	Bacteria	Proteobacteria	Rhizobiaceae	*Rhizobium*	Rhizobium etli	7568	119	119	100.00%
L33688.1	Bacteria	Proteobacteria	Rhodocyclaceae	*Azoarcus*	Azoarcus anaerobius str. LuFres1; DSM 12081	7674	25	25	100.00%
X80996.1	Bacteria	Proteobacteria	Rhodospirillaceae	*Oceanibaculum*	""Magnetic coccus""	1011	76	76	100.00%
Z23157.1	Bacteria	Proteobacteria	Sphingomonadaceae	*Sphingomonas*	Sphingomonas asaccharolytica	7035	40	40	100.00%
D16148.1	Bacteria	Proteobacteria	Sphingomonadaceae	*Sphingobium*	[Sphingomonas] chungbukensis	7440	37	37	100.00%
AF116181.1	Bacteria	Proteobacteria	Sphingomonadaceae	*Sphingomonas*	Sphingomonas phyllosphaerae str. FA1	6650	31	31	100.00%
U50711.1	Bacteria	Synergistetes	Synergistaceae	*Anaerobaculum*	Anaerobaculum thermoterrnum	512	52	52	100.00%
AJ241001.1	Bacteria	Proteobacteria	Syntrophaceae	*Desulfobacca*	Norway:(Svalbard)Hornsund clone Sva0485	10380	76	76	100.00%
Y18182.1	Bacteria	Firmicutes	Thermoanaerobacteraceae	*Thermoanaerobacter*	Clostridium uzonii str. DSM 9752	2400	54	54	100.00%
M38637.1	Archaea	Euryarchaeota	Thermoplasmataceae	*Thermoplasma*	Thermoplasma acidophilum	2178	34	34	100.00%
X86690.1	Bacteria	Firmicutes	Veillonellaceae	*Dendrosporobacter*	Dehalobacterium formicoaceticum	397	81	81	100.00%
AF287783.1	Bacteria	Firmicutes	Veillonellaceae	*Anaeroglobus*	Anaeroglobus geminatus str. AIP313.00; CIP 106856; CCUG 44773	761	55	55	100.00%
AB092894.1	Bacteria	Firmicutes	Veillonellaceae	*Dendrosporobacter*		2432	35	35	100.00%
AJ401116.1	Bacteria	Verrucomicrobia	Verrucomicrobiaceae	*Verrucomicrobium*	Elbe river clone DEV009	219	41	41	100.00%
X72876.1	Bacteria	Firmicutes	Bacillaceae	*Alkalibacillus*	Bacillus haloalkaliphilus str. DSM 5271T	3913	82	81	98.78%
AB049340.1	Bacteria	Firmicutes	Peptococcaceae 1	Peptococcaceae 1	Desulfitobacterium frappieri str. TCP-	212	55	54	98.18%
AF227158.1	Bacteria	Proteobacteria	Rhodobacteraceae	*Pannonibacter*	Pannonibacter phragmitetus C6/19	6912	52	51	98.08%
D12786.1	Bacteria	Proteobacteria	Rhodocyclaceae	*Shinella*	Mycoplana dimorpha	7051	82	80	97.56%
D14503.1	Bacteria	Proteobacteria	Rhizobiaceae	*Rhizobium*	Agrobacterium tumefaciens str. C58 Cereon	6964	118	115	97.46%
X87273.1	Bacteria	Proteobacteria	Bradyrhizobiaceae	*Bradyrhizobium*	Bradyrhizobium japonicum HA1	7087	39	38	97.44%
X68388.2	Bacteria	Proteobacteria	Rhizobiaceae	*Ensifer*	Sinorhizobium fredii	6683	112	109	97.32%
U69637.1	Bacteria	Proteobacteria	Bradyrhizobiaceae	*Bradyrhizobium*	Bradyrhizobium japonicum str. USDA 38	7398	37	36	97.30%
AF095928.1	Bacteria	Proteobacteria	Bradyrhizobiaceae	*Rhodopseudomonas*	Afipia genosp. 4 str. G3644	7333	35	34	97.14%
AF029227.1	Bacteria	Proteobacteria	Enterobacteriaceae	*Tatumella*	Salmonella bongori str. JEO 4162	8430	35	34	97.14%
U87759.1	Bacteria	Proteobacteria	Bradyrhizobiaceae	*Afipia*	Afipia genosp. 10 str. G8996	7390	31	30	96.77%
X74710.1	Bacteria	Proteobacteria	Vibrionaceae	*Vibrio*	Vibrio aestuarianus str. 01/151	8888	121	117	96.69%
D21222.1	Bacteria	Proteobacteria	Shewanellaceae	*Shewanella*	Shewanella benthica str. DB21MT-2	8581	85	82	96.47%
AJ296568.1	Bacteria	Proteobacteria	Cystobacteraceae	*Cystobacteraceae*	uranium mining mill tailing clone GR-296.II.52 GR-296.I.52	594	27	26	96.30%
M59062.1	Bacteria	Proteobacteria	Erythrobacteraceae	*Erythrobacter*	Lutibacterium anuloederans str. LC8	7036	27	26	96.30%
AF418170.1	Bacteria	Proteobacteria	Desulfovibrionaceae	*Desulfovibrio*	Desulfovibrio giganteus str. DSM 4370	10248	51	49	96.08%
AJ306754.1	Bacteria	Firmicutes	Clostridiales XII	*Acidaminobacter*	DCP-dechlorinating consortium clone SHA-58	2729	72	69	95.83%
AB089101.1	Bacteria	Proteobacteria	Rhodocyclaceae	*Azoarcus*	termite gut homogenate clone Rs-B77 proteobacterium	7824	24	23	95.83%
AF078764.1	Bacteria	Proteobacteria	Comamonadaceae	*Acidovorax*	Acidovorax delafieldii str. ATCC 17505	8018	47	45	95.74%
AF191738.1	Bacteria	Proteobacteria	Rhizobiaceae	*Ensifer*	Ensifer adhaerens str. LMG 20582	6972	117	112	95.73%
AF078765.1	Bacteria	Proteobacteria	Comamonadaceae	*Acidovorax*	Acidovorax facilis str. CCUG 2113	8021	45	43	95.56%
AJ002139.1	Bacteria	Proteobacteria	Bartonellaceae	*Bartonella*	Bartonella henselae str. Houston-1	7634	111	106	95.50%
U15102.1	Bacteria	Proteobacteria	Helicobacteraceae	*Sulfurimonas*	hydrothermal vent clone PVB_10	10543	22	21	95.45%
U81775.2	Archaea	Euryarchaeota	Methanobacteriaceae	*Methanobacterium*	Methanobacterium curvum	2285	22	21	95.45%
AF084835.1	Bacteria	Proteobacteria	Enterobacteriaceae	*Escherichia/Shigella*	USA:New York isolate str. KN4	8742	208	198	95.19%
AF078755.1	Bacteria	Proteobacteria	Comamonadaceae	*Simplicispira*	Aquaspirillum metamorphum str. DSM 1837	7807	62	59	95.16%
NC_000913.2	Bacteria	Proteobacteria	Enterobacteriaceae	*Escherichia/Shigella*		9496	241	229	95.02%
M59064.1	Bacteria	Proteobacteria	Caulobacteraceae	*Brevundimonas*	Brevundimonas diminuta	6909	20	19	95.00%
AB008503.1	Bacteria	Proteobacteria	Oxalobacteraceae	*Collimonas*	Collimonas fungivorans str. Ter331	7921	20	19	95.00%
U85862.1	Bacteria	Proteobacteria	Pseudoalteromonadaceae	*Pseudoalteromonas*	Pseudoalteromonas ruthenica str. KMM300	9324	20	19	95.00%
AB021344.1	Bacteria	Proteobacteria	Comamonadaceae	*Acidovorax*	Acidovorax sp. str. OS-6	7987	79	75	94.94%
M95665.1	Bacteria	Proteobacteria	Methylocystaceae	*Methylosinus*	Methylosinus sporium	7219	37	35	94.59%
AJ532683.1	Bacteria	Proteobacteria	Methylocystaceae	*Methylosinus*	uranium mill tailings clone Gitt-KF-194	7199	36	34	94.44%
AJ543434.1	Bacteria	Proteobacteria	Comamonadaceae	*Acidovorax*	nephridia Octolasion lacteum clone OI2-2	8152	89	84	94.38%
AF078762.1	Bacteria	Proteobacteria	Comamonadaceae	*Acidovorax*	Acidovorax avenae subsp. cattleyae str. NCPPB 961 subsp.	8022	104	98	94.23%
AB089113.1	Bacteria	Proteobacteria	Hydrogenimonaceae	*Hydrogenimonas*	termite gut homogenate clone Rs-H40 proteobacterium	10590	49	46	93.88%
U92197.1	Bacteria	Proteobacteria	Enterobacteriaceae	*Escherichia/Shigella*	Salmonella subsp. enterica serovar Agona str. Sa1 subsp.	8974	210	195	92.86%
AY221081.1	Bacteria	Proteobacteria	Nitrosomonadaceae	*Nitrosospira*	Mammoth cave clone CCU25	7808	41	38	92.68%
AJ537466.1	Bacteria	Proteobacteria	Comamonadaceae	*Ottowia*	Ottowia thiooxydans str. K11	7995	68	63	92.65%
AB015517.1	Bacteria	Proteobacteria	Helicobacteraceae	*Sulfurovum*	hydrothermal vent 9 degrees North East Rise Pacific Ocean clone CH5_6_BAC_16SrRNA_9N_EPR	10530	26	24	92.31%
AY133064.1	Bacteria	Proteobacteria	Hydrogenophilaceae	*Thiobacillus*	TCE-contaminated site clone ccs265	7817	65	60	92.31%
AB002653.1	Bacteria	Proteobacteria	Sphingomonadaceae	*Blastomonas*	Sphingopyxis flavimaris str. SW-151	6663	13	12	92.31%
AB074730.1	Bacteria	Proteobacteria	Xanthomonadaceae	*Fulvimonas*	Dyemonas todaii str. XD10	8689	13	12	92.31%
Z76664.1	Bacteria	Proteobacteria	Pseudomonadaceae	*Pseudomonas*	Pseudomonas fulva str. IAM 1587	8553	38	35	92.11%
X67224.1	Bacteria	Proteobacteria	Rhizobiaceae	*Rhizobium*	Rhizobium tropici	6770	76	70	92.11%
AB059264.1	Bacteria	Proteobacteria	Ferrimonadaceae	*Paraferrimonas*	Shewanella sp. str. MTW-1	9081	88	81	92.05%
X76564.1	Bacteria	Actinobacteria	Brevibacteriaceae	*Micrococcineae*	Brevibacterium iodinum	1745	25	23	92.00%
AJ240975.1	Bacteria	Proteobacteria	Desulfobacteraceae	*Desulfonema*	Desulfobacterium cetonicum str. DSM 7267 oil recovery water	10046	25	23	92.00%
AF441730.1	Bacteria	Proteobacteria	Rhodocyclaceae	*Shinella*	India: Himalayas Kaza Spiti Valley Cold Desert isolate str. Kaza-35 Kaza-35	6974	100	92	92.00%
X68174.1	Bacteria	Firmicutes	Clostridiaceae 1	Clostridiaceae 1	Clostridium sardiniense str. DSM 600	4598	136	125	91.91%
X72724.1	Bacteria	Proteobacteria	Burkholderiales_incertae_sedis	*Methylibium*	Anoxobacterium dechloraticum	7965	12	11	91.67%
AF170358.1	Bacteria	Proteobacteria	Pseudomonadaceae	*Pseudomonas*	Pseudomonas sp. str. KNA6-5	8777	72	66	91.67%
D14510.1	Bacteria	Proteobacteria	Rhizobiaceae	*Rhizobium*	Rhizobium huautlense	7041	120	110	91.67%
Y10011.2	Archaea	Euryarchaeota	Archaeoglobaceae	*Archaeoglobus*	Archaeoglobus lithotrophicus str. TF2	2138	35	32	91.43%
AB088961.2	Bacteria	Firmicutes	Clostridiales XIII	*Anaerovorax*	municipal wastewater treatment plant clone 012C11_B_SD_P15	3112	23	21	91.30%
AY216453.1	Bacteria	Proteobacteria	Desulfobacteraceae	*Desulfatirhabdium*	temperate estuarine mud clone KM62	9818	57	52	91.23%
AJ007802.1	Bacteria	Proteobacteria	Caulobacteraceae	*Brevundimonas*	Brevundimonas intermedia str. MBIC2712 ATCC15262	6781	22	20	90.91%
AF028688.1	Archaea	Euryarchaeota	Methanobacteriaceae	*Methanobrevibacter*	Methanobacterium bryantii	2272	11	10	90.91%
AJ431240.1	Bacteria	Spirochaetes	Spirochaetaceae	*Spirochaeta*	Spirochaeta sp. str. BHI80-158	6459	22	20	90.91%
AF003374.1	Bacteria	Proteobacteria	Rhizobiaceae	*Rhizobium*	Rhizobium sp. str. SH19312	6804	141	128	90.78%
AF067960.1	Bacteria	Proteobacteria	Pseudomonadaceae	*Pseudomonas*	Pseudomonas stutzeri str. KC	8852	65	59	90.77%
AY177804.1	Bacteria	Proteobacteria	Desulfobulbaceae	*Desulfurivibrio*	Antarctic sediment clone LH5_30	9784	42	38	90.48%
AJ344134.1	Bacteria	Bacteroidetes	Flavobacteriaceae	*Winogradskyella*	Psychroserpens burtonensis str. S2-64	5914	21	19	90.48%
AF407381.1	Bacteria	Proteobacteria	Comamonadaceae	*Rhodoferax*	Arctic sea ice ARK10281	7986	72	65	90.28%

AB089097.1	Bacteria	Proteobacteria	Hyphomicrobiaceae	*Zhangella*	termite gut homogenate clone Rs-M62 proteobacterium	7156	41	37	90.24%
AJ227782.1	Bacteria	Proteobacteria	Caulobacteraceae	*Brevundimonas*	Brevundimonas bacteroides str. CB7	7359	20	18	90.00%
AB008392.1	Bacteria	Proteobacteria	Caulobacteraceae	*Brevundimonas*	Brevundimonas subvibrioides str. CB81	7366	20	18	90.00%
Y17665.1	Bacteria	Proteobacteria	Enterobacteriaceae	*Enterobacter*	Enterobacter cloacae Nr. 3	8528	160	144	90.00%
AF524861.1	Bacteria	Proteobacteria	Rhodospirillaceae	*Telmatospirillum*	sphagnum peat bog clone K-5b5	7400	40	36	90.00%
